# Patterns in biocrust recovery over time in semiarid southeast Spain

**DOI:** 10.3389/fmicb.2023.1184065

**Published:** 2023-06-15

**Authors:** Consuelo Rubio, Roberto Lázaro

**Affiliations:** Estación Experimental de Zonas Áridas (CSIC), Almería, Spain

**Keywords:** biocrusts, recovery, dissimilarity, succession, microclimate, colonization attempts, long time series

## Abstract

Biological soil crusts (biocrusts) are communities of microorganisms, fungi, algae, lichens and mosses inhabiting on the soil surface and within the uppermost soil millimetres. They play an important ecological role in drylands, determining physical and chemical soil properties and reducing soil erosion. Studies on biocrust natural recovery establish highly variable recovery times. The different objectives and methodologies of experimentation and analysis, strongly influence these predictions. The main purpose of this research is to analyze the recovery dynamics of four biocrust communities and their relationship with microclimatic variables. In 2004, in Tabernas Desert, some of us removed the biocrust in central 30 cm × 30 cm area of three 50 cm × 50 cm plots in each of four biocrust communities (Cyanobacteria, Squamarina, Diploschistes, and Lepraria), installing a microclimatic station in each one with sensors for temperature and humidity of the soil and air, dew point, PAR and rain. Yearly, the 50 cm × 50 cm plots were photographed, and the cover of every species was monitored in every 5 cm × 5 cm cell of a 36-cells grid covering the removed central area. We analyzed different functions to fit the cover recovery, the differences in cover recovery speed between communities, the recovery dynamics from the spatial analysis of the plot, the changes in dissimilarity and biodiversity and the possible relationships with the climatic variables. The recovery of the biocrust cover fits to a sigmoidal function. The community dominated by Cyanobacteria developed faster than those dominated by lichens. The Squamarina and Diploschistes communities recovered faster than that of Lepraria and appears to be influenced by the surrounding undisturbed areas. Species-based dissimilarity between consecutive inventories fluctuated and decreased over time, while biodiversity increases in a similar way. The speed of recovery of the biocrust in each community, along with the order in which the species appeared, support the hypothesis about the succession, which would include three phases: firstly Cyanobacteria, then Diploschistes and/or Squamarina and finally Lepraria. The relationship between biocrust recovery and microclimate is complex and this work highlights the need to carry out further research on this topic and on biocrust dynamics in general.

## Introduction

Biocrusts are communities of photoautotrophic and heterotrophic organisms living in or on the uppermost millimeters of soil, which develop in close association with soil particles (Weber et al., 2022). Despite their small size, these communities can influence physical, chemical, and biological processes where they colonize. They fix atmospheric carbon (Grote et al., 2010; Sancho et al., 2016) and nitrogen (Belnap, 2002; Castillo-Monroy et al., 2010), determine the availability of nutrients and soil fertility (Mager, 2010; Miralles et al., 2012; Ferrenberg et al., 2017) and influence soil moisture (Chamizo et al., 2013; Xiao et al., 2014), infiltration, and runoff (Chamizo et al., 2016).

Biocrust plays an important role in arid and semiarid areas. The low water availability in these areas hinders the growth of vascular plants (Maestre et al., 2016) and favors the development of these communities, which can resist prolonged droughts due to their poikilohydric condition. The close relationship with soil particles allows biocrusts to act as soil stabilizers (Belnap and Büdel, 2016) and avoid the erosion (Chamizo et al., 2012a,^2017^) typical of these areas. In this way, these communities favor the increase of biodiversity and productivity (Maestre et al., 2011), favoring, in some cases, the development of vascular vegetation (Havrilla et al., 2019). In general, biocrusts provide arid and semiarid areas ecosystem services relevant to society (Rodríguez-Caballero et al., 2018a). However, despite their apparent stability, the organisms of these communities, mainly lichen and mosses, are fragile. Changes in temperature, precipitation, or land use and disturbances such as trampling or grazing reduce biocrust cover significantly (Maestre et al., 2013; Ferrenberg et al., 2015; Rodriguez-Caballero et al., 2018b) and increase erosion, favoring the degradation of these areas (Belnap, 1995; Cantón et al., 2021). The main threats to biocrusts are probably mechanical destruction by trampling, off-road vehicles or livestock, or land use change, when the ecosystem ceases to be natural. Soil and air pollution can also affect them. According to Rodriguez-Caballero et al. (2018b), biocrusts cover about 12.2% of the Earth’s surface, but it could be reduced between 25 and 40% in 2070 as a result of human activity and disturbance.

Concern for the survival of these communities has led many researchers to determine how they can recover and how long they take to recover (Cantón et al., 2021). Researchers have studied biocrust restoration through inoculation with Cyanobacteria (e.g., Román et al., 2018, 2021) with promising results. Other researchers have tried to restore biocrusts through organism translocation (e.g., Belnap, 1993; Davidson et al., 2002; Bowker et al., 2010); however, that technique does not allow the complete recovery of the biocrust and may compromise its integrity in other areas (Zhao et al., 2016). Finally, restoring biocrusts using cultivated organisms, although potentially successful for moss (Doherty et al., 2020), seems infeasible for lichen biocrusts, which are widespread in semiarid areas. Considering this difficulty, parallel studies on natural recovery are important, because they could serve as a basis for the management of degraded areas and because any possibility of accelerating recovery requires knowing the natural process. However, little is known about how recovery works, and estimated recovery times are highly variable between studies. For arid and semi-arid environments, recovery time ranges from less than 3 years for cyanobacterial crust after a biocrust removal (Dojani et al., 2011) to 2000 years for lichen recovery after tank tracks are made (Belnap and Warren, 1998, 2002).

In 2001, Belnap and Eldrige (and later, Weber et al., 2016), conducted a review of biocrusts’ natural recovery and concluded that recovery is dependent on physico-chemical and climatic conditions as well as the type and the severity of disturbance; they also highlighted methodological problems, such as the visual estimation of coverage, the number of observations, or the use of linear extrapolations to predict recovery time. In 2020, Kidron et al., 2020 carried out a new review and highlighted the lack of analysis of other variables, such as the measurement of chlorophyll or biomass. This wide variability of influencing factors and of research viewpoints makes it difficult to correctly estimate the recovery times of various biocrust components and to determine the best experimental design for this type of study. A reassessment of articles on natural recovery might indicate that the main problem to reach consistent results about natural recovery processes is that most researchers do not focus on how the recovery occurs. Around 90% of the works have the objective of determining the capacity and recovery time of biocrusts after various disturbances, using different methodological approaches and based only on a few observations (Johansen et al., 1984; Lalley and Viles, 2008; Langhans et al., 2010; Williams et al., 2018, among others), which overestimates the calculated recovery times (Belnap and Eldridge, 2001) and make it difficult to establish reliable recovery times.

It is opportune to point out that recovery studies, by definition, monitor secondary succession, which, in principle, could be different from primary succession -at least, under some environmental conditions. We have not found long-term monitoring of primary succession in biocrusts in the literature to examine this possible difference.

The purpose of this research was to analyze the recovery dynamics of four biocrust communities and their relationship with microclimatic variables. To achieve this, *in situ* recovery plots were monitored for 17 years at the El Cautivo experimental area, Tabernas Desert, semiarid southeast Spain. These four communities, described below, account for almost all the biocrust variation in the field site, and have received attention from various researchers (Rodríguez-Caballero et al., 2013; Cantón et al., 2014; Chamizo et al., 2015; Grishkan et al., 2019; Miralles et al., 2020; among others). Concrete objectives were establishing how recovery starts, differences in recovery among communities, which functions best explain the evolution of biocrust covers over time, and what microclimatic factors influence recovery. The first results of this monitoring were published by Lázaro et al. (2008), and following that publication, we assume that the monitored biocrusts can be treated as communities because they have a differentiated and fairly stable composition over time, a composition that would change slowly. We hypothesize that these communities are representative of three stages of succession, because these authors provide evidence both for replacement between species and for varying recovery speeds of different biocrust communities. Due to this, in this research we discuss the results based on the assumption that the monitored communities may be in stages of succession in which the habitat allows the evolution of the biocrust. Thus, we hypothesize that succession does not occur equally at every community, as each one is associated with certain ranges of landforms and microclimate (Lázaro et al., 2000) and is stable enough over time, according to our own field observation during the last 34 years: The cyanobacterial community would be only preceded by incipient cyanobacterial biocrust and would be virtually permanent in the sunniest site where the monitoring plots are. The Squamarina and Diploschistes communities would be two facies of the same successional stage, both preceded by that of cyanobacteria, and would be practically permanent in the sampled slopes as well as in much of the study area slopes. The full succession could occur in the shadiest slopes of the Lepraria community, starting with light or dark Cyanobacteria, followed by Squamarina and/or Diploschistes communities, and culminating with the typical Lepraria community.

## Materials and methods

### Study site

The Tabernas Desert is located to the north of the city of Almeria, in the southeast of Spain. The landscape is characterized by extensive badlands because of erosion, and the climate is semiarid warm Mediterranean. The mean annual precipitation is about 230 mm, and the mean annual temperature is 18°C, with a maximum and minimum of 45 and −5.5°C, respectively (Lázaro et al., 2001, 2004).

The patchy vegetation, dominated by tussock grasses, dwarf shrubs, and annual herbs, is concentrated in locations favored by orientation and runoff. It includes a high proportion of individuals belonging to aridity-adapted species, having Iberian/North African distributions, as well as some endemisms. The biocrusts spread through the spaces between patches as well as the areas where vascular plants cannot develop, as long as these areas are not undergoing erosion (Lázaro et al., 2000). For more information about the study area, see Calvo-Cases et al. (2014). In this study area, landforms generate various types of microhabitats in which different communities of biocrusts appear. Four communities have been identified, and they were defined by the predominant or characteristic lichen species. The cover and species composition of these biocrusts are stable enough, at least in contrasting microhabitats, to be considered communities (Lázaro et al., 2008). However, they are not exempt from being affected by changes in the conditions of the microhabitat that might favor the evolution to a next successional stage (Deng et al., 2020). In fact, Lázaro et al. (2008) provided evidence on the replacement of species. In the same way, communities can also return to a previous successional stage in the face of environmental changes that harm their development (Ferrenberg et al., 2015). The communities in this study were chosen as good representations of the microclimates and sufficient contrast in the composition of species. The finding of other communities in the area would be possible because the composition is intermediate in the microclimatic ecotones, but the ecotones occupy relatively little space. Finally, we considered the monitored biocrusts both as communities and as categories or stages in a hypothetical successional gradient, and we define them below ordered from the earliest to the latest.

- Cyanobacteria extends over vast insolated plains and is dominated by a microbial crust mainly composed by Cyanobacteria (21.89%), but also by Bacteroidetes (14.25%), Proteobacteria (13.15%), Actinobacteria (9.84%), and Chloroflexi (9.67%), among others (Miralles et al., 2020). Some small lichens, including *Endocarpon pusillum* Hedw, *Fulgensia desertorum* (Tomin) Poelt, *Fulgensia poeltii* Llimona, and *Fulgensia fulgida* (Nyl.) Szatala are also characteristic of this community, which is named for its high proportion of Cyanobacteria, along with the fact that this bacterial phylum decreases significantly through the succession.
- Squamarina is the most extended community and dominates most of the slopes of the experimental area. It develops on a microbial crust, visible in the spaces between lichens. It is dominated by the lichen *Squamarina lentigera* (Weber) Poelt, although other species of lichens are also frequent, such as *Buellia zoharyi* Galun or *Diploschistes diacapsis* (Ach.) Lumbsch.
- Diploschistes grows closely with the Squamarina community, sometimes interspersed, but the lichens *Diploschistes diacapsis* (Ach.) Lumbsch and *Diploschistes ocellatus* (Vill.) Norman are dominant, which give it a rough appearance. Other species such as *Buellia zoharyi* Galun, *Squamarina lentigera* (Weber) Poelt or *Fulgensia fulgida* (Nyl.) Szatala are also frequent.
- Lepraria extends over the shadiest slopes, where vascular vegetation is relatively abundant (30–40% cover) compared to the rest of the communities. It is the most diverse community and is characterized, but not necessarily dominated, by more mesic lichens such as *Lepraria isidiata* (Llimona) Llimona & Crespo (species that gives it its name) and others such as: *Squamarina cartilaginea* (With.) P. James, *Xanthoparmelia pokornyi* (Körb.) O. Blanco, A. Crespo, Elix, D. Hawksw. & Lumbsch and *Teloschistes lacunosus* (Rupr.) Savicz. On the other hand, although the moss cover is the minority, moss species are more frequent in this community, highlighting the presence of *Grimmia pulvinata* (Hedw.) Sm. Other lichen species, including those that characterize the Squamarina and Diploschistes communities, are also frequent. These lichens develop on a matrix of microbial biocrust that is darker than that in the rest of communities, and phyla such as Proteobacteria, Bacteroidetes and Planctomycetes are much more frequent, accounting for near 50% of bacteria together, whereas Cyanobacteria only represents c. 2% here (Miralles et al., 2020).

### Experimental design and data collection

In 2004, twelve plots of 50 cm per side were established, equally distributed over the four communities found in the Tabernas Desert (Cyanobacteria, Squamarina, Diploschistes, and Lepraria). We assumed that the plots were of sufficient size to constitute an acceptable representation of every community (Eldridge and Greene, 1994), and that, in these conditions and spatial scale, recovery drives toward a community composition statistically similar to that of the original community. In each plot, the biocrust of the central 30-cm-side area was removed, leaving the surrounding area of 10 cm intact. The entire thickness of the biocrust was removed, taking care to minimize the removal of the underlying soil so that biocrust recovery occurred under conditions as similar as possible to those of the original biocrust. The depth of the removal was variable in space; at each point, we attempted to remove the minimum that ensured that elements of biocrust were no longer seen with the naked eye. We disturbed all plots with the same intensity precisely because, if the communities constitute a succession, it is expected that some of them take longer to recover. On the other hand, any disturbing pattern other than applying the same alteration to all communities would be arbitrary and difficult to justify.

Photographs of the complete plots were taken annually from approximately 1 m high, although the main criterion was to include the four corner marks of the undisturbed area. Species inventories were also carried out annually (except for 4 years, from 2015 to 2018), in the disturbed area. For the inventories, a grid of 30 cm (of the same area as the removed biocrust) divided into 36 5-cm-side cells was used. The cover of every lichen species, expressed in mm^2^, was estimated for each grid cell. Light and dark microbial crusts (which are easily distinguishable from bare soil by their colors) were considered as two additional species; the moss *Grimmia pulvinata* was recorded separately and individuals of any other moss species (which are small, scarce, often annual, and very difficult to identify with the naked eye) were recorded as “moss”. The extent covered by bare soil was also recorded.

Because this experiment lasted longer than initially imagined and because field observation suggested differences over time in the biocrust of the undisturbed peripheral areas of the plots, the cover in the intact area of the plots was calculated using the transect method from the 2006 and 2021 images to analyze how those areas had changed through the experimental period. To do that, we digitized four transects of 50 cm on the photographs of each of the plots, one per each side of the undisturbed perimeter, using ArcGIS 10.5 (Esri Inc, 2016, USA). Along transects, the space occupied by the microbial crust, lichen crust, mosses, and bare soil was digitized with a resolution of 1 mm. The cover of each component was obtained compared to the total set of transects of the plot; that is, for any surface category, % cover = (sum of lineal mm × 100)/2,000.

### Microclimatic data

Automatic weather stations were set up in 2004 at each of the described communities, configured to collect data every 20 minutes. Air temperature and relative humidity were measured by an S-THB-M00x sensor (Onset, USA). Soil temperature was measured by an S-TMB-M0xx sensor (Onset, USA). Soil water content was measured by an S-SMA-M00x sensor (Onset, USA), and photosynthetically active radiation (PAR) was measured by an S-LIA-M00x sensor (Onset, USA). Rainfall was measured by the Rain-O-Matic-Pro tipping-bucket rain gauge of 0.25 mm resolution (Pronamic, Denmark).

The data obtained from the microclimatic stations were processed by removing the wrong data and filling in the gaps using the R package “mice” (van Buuren and Groothuis-Oudshoorn, 2011; R Core Team, 2020, Austria). To do this, part of the database of the different climatic variables (involving each microclimatic station) that did not have gaps and was large enough to define the daily fluctuations was selected. In this selection, random gaps were generated using the “missForest” package and then filled with various combinations of imputation models and number of iterations using the “mice” function. The results of each of the combinations were compared with the originals of the selected part of the database, and the coefficient of determination (R^2^) and the root-mean-square error (RMSE) were calculated to select the best imputation model for each variable. Finally, the selected imputation model was applied to the entire database of each of the climatic variables to generate a complete database for each station. From the complete databases, the number of records having potential dew (when biocrust surface temperature was lower than the calculated dewpoint), the number of records having soil water content higher than 10%, and the number of rain days were calculated.

### Data analysis

Due to the differences in development observed in the field and by other authors (Belnap and Eldridge, 2001; Dojani et al., 2011; Lorite et al., 2020), we grouped the species cover from the inventories in two general biocrust-type covers present in all the communities analyzed: microbial crust, composed of light and dark crust, and lichen crust, composed of lichen and moss species. Mosses were not considered separately due to their much lower abundance. We used these two biocrust-types covers as dependent variables in the analyses (detailed later).

To analyze the dynamics of recovery and growth in each community, six types of growth functions (polynomial, sigmoidal, exponential, rational, power and logarithmic) and their variants (10 functions in total) were fitted to the changes in the cover over time using SigmaPlot 14.5 (Systat Software Inc, 2020, USA). For each of the communities and biocrust types (microbial crust and lichen crust), the Akaike Information Criterion (AIC) and the coefficient of determination (R^2^) of each function were obtained, and the function that best fit the distribution of the plot data was determined. Low AIC values and high R^2^ values indicate a better fit to the data to the function.

The differences between communities throughout the studied period for each of the biocrust types were analyzed through generalized linear mixed models (GLMMs). We used plot cover data to conduct this analysis, defining community as a between-subject factor and time after disturbance as a within-subject factor.

The spatial patterns of recovery were analyzed by examining the distribution of the emergent organisms regarding a series of rings defined on the surface of the plot. These rings are the result of groups of inventoried cells based on the proximity to the control areas. Thus, the outer ring is composed of the cells of the outer perimeter of the grid, the middle ring is composed of the cells of the intermediate area of the grid, and the center ring is made up of the four central cells of the grid. Differences in recovery between communities and rings for microbial and lichen crust were analyzed using generalized linear mixed models (GLMM). Cell-scale cover data was used for this analysis, defining community and rings as a between-subject factors, and time after disturbance as a within-subject factor.

Based on the experience of the field inventories, we assumed that numerous colonization attempts were not successful (because a certain species disappeared from a certain cell of the grid and perhaps appeared in another, regardless its changes in cover), and that, as recovery progressed, the composition of the community would change less. Therefore, in addition to cover, the stability of the biocrust composition over time would be a criterion for deciding whether the biocrust had recovered. We used the rate of change of the community composition over time, analyzed through the Bray–Curtis distance (Bray and Curtis, 1957; Somerfield, 2008) as an index of the recovery of biocrust composition. Plots were compared with themselves over time, considering species cover as variables and considering the light and dark microbial crust separately as two species. The Bray–Curtis index was selected because (i) it can only take values in the range from 0 to 1, which facilitates comparisons; (ii) equal absolute differences between values of a variable do not have the same weight through the entire range of values of the variable; they have more weight the closer they are to the maximum values. This seems adequate because a cover difference of 0 to 5 is more significant in terms of recovery than one of 20 to 25; and (iii) the Bray–Curtis algorithm does not take double absences into account when comparing, which, in addition to being correct (otherwise, the inventories of different years could be more similar due to many species that neither of them have, as opposed to diverging due to the changes in the ones they have) facilitates computation, making it unnecessary to remove the fields corresponding to variables with double zero value.

We also analyzed changes in biodiversity through the Shannon–Wiener index (Shannon, 1948) from the species cover data (including the light and dark microbial crust, *Grimmia pulvinata* and mosses as separate species) for each year and each plot. Differences between communities for the Bray–Curtis distance and Shannon–Wiener index were analyzed using GLMMs. For Bray–Curtis analysis, we used cell-scale dissimilarity data, and defined community as a between-subject factor and time after disturbance as a within-subject factor. For the Shannon–Wiener index analysis, we used plot diversity data, and defined community as a between-subject factor and time after disturbance as a within-subject factor. Because the biocrust was removed at the beginning of the experiment (time 0), we assumed a Bray–Curtis distance of 1 and a Shannon–Wiener index of 0 for this initial time point in our analyses. All the GLMM analyses were made using SPSS 28.0 (IBM Corporation, 2021, USA).

The changes in microbial and lichen crust cover were related to microclimatic variables through multiple regressions. Finally, because the habitats of the various communities show microclimatic differences, and most of the microclimatic variables are related, synthesizing the microclimatic variables in a few components provided information on the environmental preferences of the biocrust communities analyzed. Thus, a multivariate method based on Principal Component Analysis (PCA) was used to display the microbial and lichen crust covers in the space of the two principal axes defined by eight microclimatic variables (mean air temperature, number of records with potential dew, mean soil surface temperature, mean soil water content, number of records with soil water content over 10%, mean PAR, total rainfall volume, and number of rain days). The PCA was conducted using Statgraphic Centurion 18 (Statpoint Technologies, Inc, 2017, USA).

## Results

### Changes over time in cover of microbial and lichen crusts in the various communities

**FIGURE 1:**
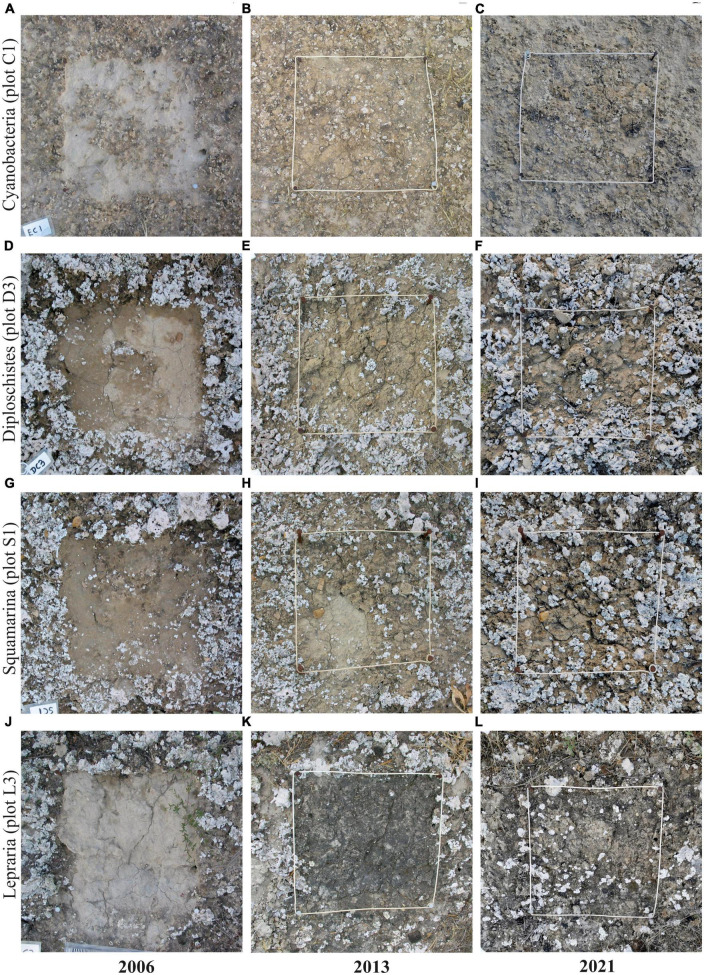
Example images of four plots that show the state of biocrust recovery in 2006, 2013, and 2021 in each of the communities analyzed: Cyanobacteria **(A–C)**; Diploschistes **(D–F)**; Squamarina **(G–I)**; and Lepraria, **(J–L)**. Photographs show the undisturbed and disturbed areas. Undisturbed areas frame the disturbed ones, which are delimited by initially equidistant nails separated 30-cm until 2009. From 2010, a 30-cm-side white wire attached to the nails delimits disturbed area and serve as a scale reference.

We found differences in recovery between the communities dominated by the microbial crust and those dominated by lichen crust, which agree with field observations (Figure 1). Figure 1 shows four randomly selected examples of the state of recovery in the first years (2006), in the middle (2013), and at the end of the experiment (2021) for each community studied. As seen in the figure, the Cyanobacteria community was fully recovered in 2013, while the other communities needed more time to recover. In particular, Lepraria, although almost completely covered by the dark microbial crust, was far from its usual appearance observed in the field as of 2021. In 2010 one of the plots (S1) was stepped on, disrupting part of the recovery achieved, which is still clearly visible in 2013: see the lower left quadrant of Figure 1H.

**FIGURE 2:**
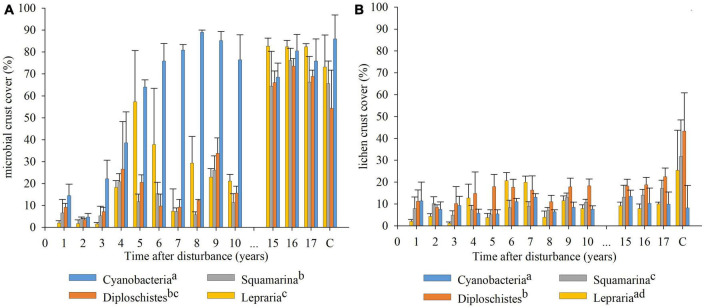
Recovery evolution of microbial crust **(A)** and lichen crust **(B)**, obtained from the annual species inventories, for each of the communities analyzed. Coverage of the control areas in 2021 (17 years after disturbance) for each community added at the end of the *x* axis. Different letters in the legend indicate significant differences between communities during the recovery of disturbed areas. (*p* < 0.05).

The GLMM result of microbial and lichen crust covers along the studied period indicated significant differences between communities (Figure 2). The Cyanobacteria community had a significantly higher microbial crust cover than the other communities; however, the lichen crust cover was significantly lower than that of Squamarina and Diploschistes and similar to that of Lepraria. The microbial crust cover was similar for Diploschistes and Squamarina, but the lichen crust cover was significantly higher in Diploschistes than in the other communities. Finally, the Lepraria community had more microbial crust cover and less lichen crust cover than Squamarina and Diploschistes. Note that Cyanobacteria community reached the maximum coverage in the first 8 years (Figure 2A). However, based on field observations, its thickness was still visibly lower than that of the surrounding control area; that difference in thickness disappeared 13 years after the biocrust removal.

After fitting the data to 10 growth functions, the R^2^ values indicated that the microbial crust was better fitted (higher R^2^) to the various functions than the lichen crust (lower R^2^) (Table 1). The results of the AIC revealed that the microbial crust was better fitted to a sigmoid function in the Cyanobacteria community, although its fit to the Gompertz distribution was not ruled out. In the Squamarina and Diploschistes communities, the recovery of the microbial crust fitted exponential growth better, but also fitted well for sigmoidal or cubic function. In the Lepraria community, microbial crust fitted better to a cubic function. Lichen crust recovery fitted better to a power function in Cyanobacteria, Squamarina and Diploschistes communities, and a cubic or sigmoidal function in the Lepraria community.

**TABLE 1:** Fits to 10 growth functions of the microbial and lichen crust cover data for each of the communities analyzed.

	Cyanobacteria		Diploschistes		Squamarina		Lepraria	
	AIC	*R* ^2^	AIC	*R* ^2^	AIC	*R* ^2^	AIC	*R* ^2^
Microbial crust								
Linear	265.25	0.53	216.70	0.77	221.76	0.75	245.56	0.70
Quadratic	214.69	0.87	203.62	0.83	202.34	0.85	245.01	0.72
Cubic	217.26	0.87	204.46	0.85	203.02	0.86	243.86	0.75
Sigmoid	190.32	0.93	202.24	0.84	200.22	0.86	244.91	0.72
Gompertz	194.93	0.92	202.80	0.84	201.65	0.85	244.88	0.72
Exponential rise to maximum	270.77	0.43	269.18	0.14	272.12	0.11	286.76	0.16
Exponential growth	274.81	0.41	200.65	0.84	199.71	0.85	242.88	0.72
Rational	236.30	0.76	203.02	0.83	226.57	0.72	246.54	0.70
Power	248.37	0.69	205.87	0.82	203.18	0.84	243.73	0.72
Logarithm	237.04	0.76	244.84	0.54	249.44	0.51	260.27	0.58
Lichen crust								
Linear	128.66	0.12	149.34	0.38	106.95	0.53	159.32	0.10
Quadratic	130.39	0.14	146.19	0.46	109.39	0.53	150.43	0.31
Cubic	132.06	0.16	143.17	0.53	110.76	0.55	150.03	0.36
Sigmoid	123.02	0.28	144.88	0.48	109.70	0.53	148.29	0.34
Gompertz	123.11	0.28	144.14	0.49	109.58	0.53	149.16	0.33
Exponential rise to maximum	188.90	NA	143.86	0.43	121.81	0.30	150.87	0.22
Exponential growth	128.99	0.12	152.01	0.34	107.70	0.53	160.35	0.07
Rational	120.50	0.28	139.07	0.52	113.88	0.45	150.90	0.26
Power	120.11	0.29	137.48	0.53	106.43	0.54	153.51	0.21
Logarithm	120.18	0.28	137.91	0.53	111.40	0.48	152.30	0.23

AIC and R^2^ values for each of the communities and crusts analyzed. All regressions are significant (*p* < 0.05).

### Biocrust recovery differences between communities regarding the proximity to the undisturbed edge

**FIGURE 3:**
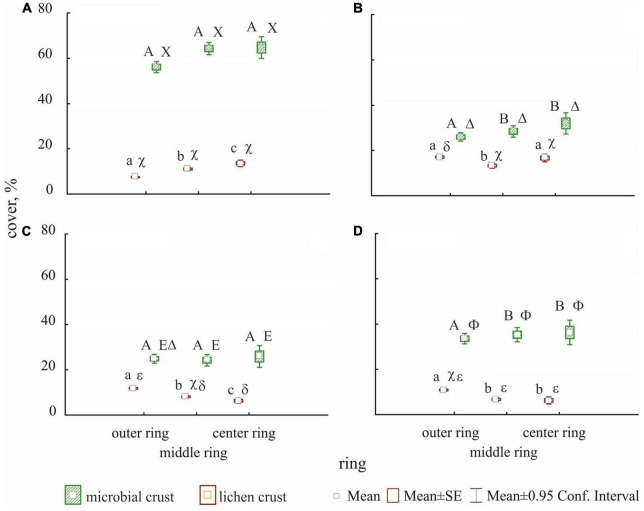
Mean covers of microbial crust (green) and lichen crust (red) of the entire sampling period, obtained from the annual inventories, showing the interaction between ring and community during recovery. Each graph shows the effect of the distance regarding the unaltered biocrust border (the ring) in a concrete community: Cyanobacteria **(A)**, Diploschistes **(B)**, Squamarina **(C)**, and Lepraria **(D)**. To show the significance according to the generalized mixed models, we used capital letters for the microbial crust cover and lowercase letters for the lichen crust cover. Latin letters (over the boxes, on left) refer to the differences among rings within every community (into each graph), whereas Greek letters (on the right) refer to the difference among communities within every ring (through the four graphs). In each pair, different letters indicate significant difference (*p* < 0.05).

The analysis of the recovery dynamics based on the analysis of the rings of cells shows significant differences between rings and between communities for the microbial and lichen crust covers (Figure 3). The outer cell ring of the plot had significantly less microbial crust cover than the central rings (middle ring and center ring) in the Diploschistes and Lepraria communities. However, no significant differences in microbial crust cover occurred between the rings in Cyanobacteria and Squamarina communities. The differences between rings were most evident for the lichen crust. The Cyanobacteria community, in contrast to the lichen-dominated communities, had less lichen crust cover in the outer ring compared to the middle and center rings, with the latter having a significantly higher lichen crust cover. In the lichen-dominated communities, the outer ring had a significantly higher lichen crust cover than the middle and center rings, except for the Diploschistes community, which showed no significant difference between the outer and central parts of the plot.

### Changes over time in dissimilarity and biodiversity

**FIGURE 4:**
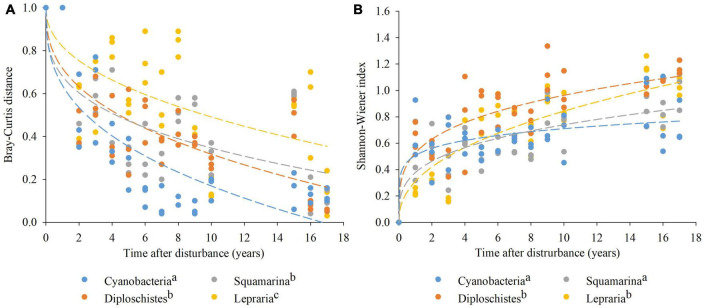
Evolution of the Bray–Curtis distance **(A)** and the Shannon–Wiener index **(B)**, obtained from the annual species inventories, for each of the communities analyzed. The points represent the results of dissimilarity and biodiversity throughout the experiment of all the plots of each community. The dashed lines represent the fits of the data to a potential function for each community. Different letters in legend indicate significant difference between communities (*p* < 0.05).

The analyses of the biocrust communities’ composition through the Bray–Curtis and Shannon–Wiener indices show changes in the composition and diversity of the communities over time. On one side, the evolution of the Bray–Curtis index decreased in all communities, showing significant differences between them (Figure 4A). The Cyanobacteria community had a lower dissimilarity index than the rest of the communities, reaching a dissimilarity close to zero after the 10th year of recovery, and zero after 17 years. Squamarina and Diploschistes communities did not have significantly different dissimilarity indices, but they did have significantly greater dissimilarity than the Cyanobacteria community and lower dissimilarity than Lepraria. The Squamarina and Diploschistes communities reached values relatively close to zero (around 0.20) 17 years after the disturbance, and the Lepraria community had higher dissimilarity indices than the rest of the communities, maintaining a value of 0.35 after 17 years of recovery.

Contrary to the Bray–Curtis index, an increasing evolution of the Shannon–Wiener biodiversity index is observed (Figure 4B). The GLMM analyses seem to indicate that the Cyanobacteria community was the least biodiverse, reaching values of 0.7 after 17 years of recovery. Squamarina did not show significant differences compared to Cyanobacteria, and it also displayed low biodiversity compared to the lichen-dominated communities. The Diploschistes and Lepraria communities reached the greatest biodiversity (significantly different from Squamarina and Cyanobacteria), obtaining values close to 1 after 10 years of recovery. However, although Lepraria’s diversity was lower than the other communities in the first years, it increased consistently more quickly than the others, reaching values of 1 17 years after disturbance.

### Relationship between the biocrust recovery and the microclimate

**FIGURE 5:**
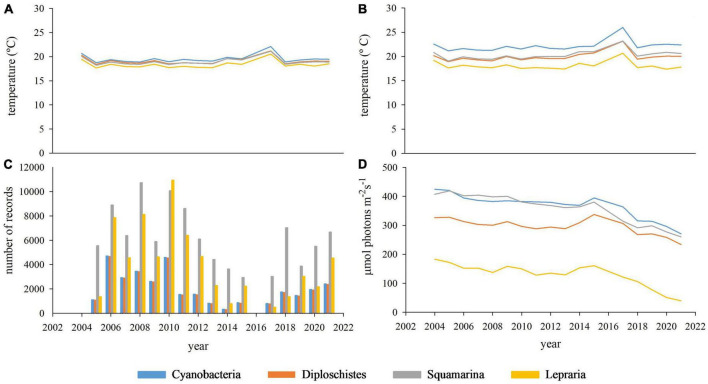
Evolution over the sampling period of the main microclimatic variables in each community: annual air temperature average **(A)**, annual soil surface temperature average **(B)**, annual number of records having soil water content over 10% **(C)**, annual photosynthetically active radiation average [PAR **(D)**].

**FIGURE 6:**
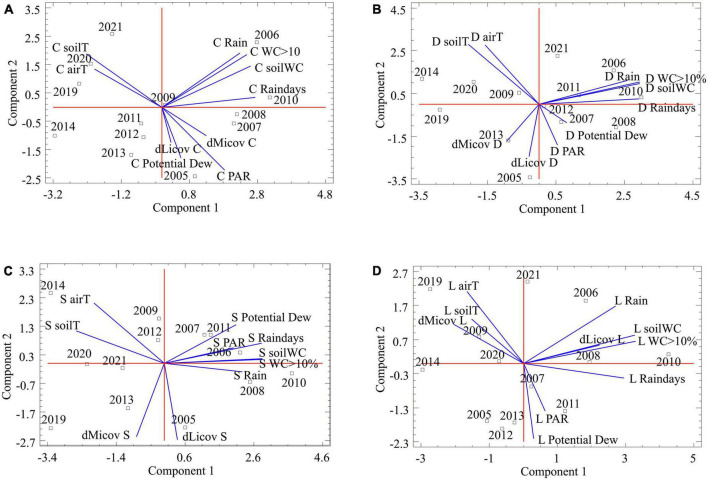
Results of the principal component analysis (PCA) to explore the relationship between the annual difference cover of microbial (dMicov X) and lichen crust (dLicov X) and the microclimatic variables. Each graph represents one community: Cyanobacteria **(A)**, Diploschistes **(B)**, Squamarina **(C)**, and Lepraria **(D)**. The microclimatic variables are: annual precipitation (Rain), number of days of precipitation (Raindays), mean soil water content (soilWC), number of records having soil water content over 10% (WC > 10), mean soil temperature (soilT), mean air temperature (airT), number of records where the soil temperature is below the dew point (Potential Dew) and photosynthetically active radiation (PAR). The letters to the right of the annual differences in coverage correspond to the initials of each of the communities: Cyanobacteria (C), Diploschistes (D), Squamarina (S), and Lepraria (L). These same letters are on the left in microclimatic variables. The numbers correspond to the years sampled.

Despite differences in most variables among microclimates/biocrust communities (see Figure 5), regressions between yearly microbial or lichen crust covers and annual climatic data were not significant. The PCA showed that two or three components can account for more than 80% of the total microclimatic variance (depending on the number and nature of the microclimatic variables involved). Figure 6 shows an example of PCAs; the microclimatic composition of the component is consistent in all the communities and the position of the microbial and lichen crust covers are plotted on the microclimatic space defined by the two main components.

Note that the recovery of the microbial and lichen crust is strongly associated with the potential dew and PAR in Cyanobacteria, and lightly associated in Diploschistes community. For Squamarina, the recovery of both biocrust types appears unassociated with any microclimatic variable, maybe because that is the most widespread community. Finally, the recovery of the lichen crust in Lepraria is strongly associated with rainfall and soil water content, but not with potential dew and PAR.

### The evolution of the control areas and the current state of recovery

The estimates of edge covers of the plots (considered the control) show a significant increase of between 10 and 20% of the microbial crust cover and a proportional and significant decrease of the lichen crust cover between 2006 and 2021 (Table 2) in all communities, except in the Lepraria community.

**TABLE 2:** Microbial and lichen crust cover of the surrounding undisturbed area in 2006 and 2021, and of the disturbed area in 2021 for each of the communities analyzed.

	Undisturbed		Disturbed
	2006	2021	2021
Microbial crust			
Cyanobacteria	78.77 @ 6.54 a	86.05 @ 7.04 bA	75.90 @ 8.05 A
Diploschistes	31.76 @ 15.78 a	54.35 @ 11.50 bA	68.99 @ 2.14 B
Squamarina	55.56 @ 10.29 a	65.7 @ 6.95 bA	66.35 @ 11.20 A
Lepraria	59.06 @ 22.96 a	73.23 @ 9.75 aA	82.44 @ 1.54 B
Lichen crust			
Cyanobacteria	15.70 @ 7.34 a	8.33 @ 5.45 bA	9.99 @ 3.89 A
Diploschistes	65.41 @ 15.97 a	43.46 @ 12.16 bA	22.60 @ 3.56B
Squamarina	43.65 @ 9.28 a	31.79 @ 9.55 bA	17.18 @ 3.58 B
Lepraria	34.28 @ 22.11 a	23.18 @ 10.78 aA	10.14 @ 0.67 A

Mean and standard deviation of the microbial and lichen crust cover of the undisturbed area in 2006 and 2021 (estimated from the digitized transects on the photographs of the plots), and of the disturbed area in 2021 (estimated from the annual species inventories). Differences in microbial and lichen crust cover were analyzed by Mann–Whitney test. Different lowercase letters indicate significant differences (*p* < 0.05) between 2006 and 2021 cover of undisturbed areas. Different capital letters indicate significant differences (*p* < 0.05) in the 2021 cover of the disturbed and undisturbed areas.

In 2021, the microbial crust of the disturbed areas reached the cover of the control areas (Figures 1, 2), and even the lichen-dominated communities had more microbial crust than in 2006 (Table 2). The lichen crust reached the same level of coverage as the control areas in the Cyanobacteria community, but not in the other communities, where the lichen crust cover was about half that of undisturbed areas (Figure 1 and Table 2).

## Discussion

Our results show differences in recovery between the community dominated by microbial crust (Cyanobacteria) and the lichen-dominated communities (Figure 2). This seems to be determined by the characteristic composition of each of the communities. While the recovery was mainly determined by the development of a microbial crust in the Cyanobacteria community, the recovery was probably limited by the colonizing capacity and the growth rate of the lichens that composed them in the communities dominated by lichens.

Several authors have found that the ability to colonize and the growth rate of microbial crust are greater than that of lichen or moss crust (Belnap and Eldridge, 2001; Dojani et al., 2011; Lorite et al., 2020; among others). In fact, this could be perhaps the only certainty to date in relation to succession in biocrusts. As already described by Belnap and Eldridge (2001) and Deng et al. (2020), microbial crust is the first colonizer in all communities and the first stage of succession. Here, this first stage would be represented by the community of Cyanobacteria, characterized by a much greater cover of microbial crust than lichen crust. The rapid recovery of this community, along with the fact that it is the only one showing larger cover in the center ring during its development, indicates that the organisms that characterize it grow quickly and have a high colonizing capacity, recovering the biocrust in the first 10–13 years after disturbance, in our case. Consistently, we also observed that the microbial crust recovered more quickly than the lichen crust in the Squamarina, Diploschistes, and Lepraria communities. The fact that microbial crust developed more quickly in any community and reached the cover it has in the undisturbed areas around (or even larger) by 2021 suggests that microbial crust is best understood as a practically all-encompassing matrix, and lichen crust develop upon or replace that matrix.

In 2004, when this recovery experiment was established, Lázaro et al. (2008) distinguished between Squamarina and Diploschistes biocrusts based on the very local composition of the community. The differences in the lichen recovery of both communities (Figure 2B) could suggest that they belong to two different successional stages. One cause of this difference could be due to differences in the structure of the thallus of the dominant species; *Diploschistes diacapsis* grows more diametrically than *Squamarina lentigera* according to Souza-Egipsy et al.’s (2002). Other cause could be the fact that one Squamarina plot suffered a shoes mark by 2010 and its effect is still clearly visible by 2013 (Figure 1H). And another possible cause could be the relationship with microclimatic variables; the recovery of the biocrust appears to be associated with potential dew and PAR in Diploschistes, but not in Squamarina (Figure 6). However, both communities recover their species composition in a similar way (Figure 4A), and they overlap greatly in the space. Thus, we think that the difference in recovery velocity is not enough to clearly indicate that they represent two successional steps. In these two communities, lichen crust grows simultaneously but more slowly than the microbial crust, replacing it (Figure 1), and their recovery is faster than that of the Lepraria community.

We understand that Lepraria is the latest in terms of succession because it takes the longest time to develop, which allows the appearance of more generalist lichen species (more euryoeic) during the process. The slow recovery of the lichen crust and the high Bray–Curtis distance in the Lepraria community (Figure 4A), compared to the others, seem to indicate difficulties in the lichen’s colonization attempts, which often were unsuccessful. These failures in colonization were observed during the experiment because the procedure used in the inventories allowed us to locate the individual small thalli that were beginning to grow, which disappeared the following year in that concrete location, whereas other new thalli could be found in other cells. In addition, the high biodiversity of this community (Figure 4B) seems to be an indicator of its ecological maturity.

These differences between communities align with the successional process proposed by Lázaro et al. (2008). Thus, the studied communities could represent various successional stages and could be used for purposes requiring space-for-time samplings. The results suggest that the lichen communities need certain microhabitat preconditions, favored by other previous organisms, to develop. In a conceptual scheme about biocrust succession in Tabernas Desert, the microbial crust would colonize first (Cyanobacteria community), favoring soil stability and increasing porosity (Miralles et al., 2011) and organic matter (Miralles et al., 2017), as well as allowing the appearance of pioneer lichens. Next, the development of the lichen crust (Squamarina and Diploschistes communities) would further improve the biological, physical, and chemical conditions of the habitat (Chamizo et al., 2012b) if the microclimate is suitable; basically, not too much insolation, to avoid the biocrust becoming dry very early in the morning and shortening the time useful for photosynthesis (Uclés et al., 2015). Note that in our experimental area, biocrust activity is higher between October and December, when solar radiation is lower (Pintado et al., 2010; Raggio et al., 2014). Finally, the improvement of habitat conditions favors the appearance of stenoic lichens (adapted to a narrow range of environmental conditions) where the microclimate is adequate, as well as of mosses and vascular plants (Lepraria community). This agrees with descriptions by Deng et al. (2020), who conclude that each of the successional stages is determined by the resources that favor the species of the previous successional stages. It is often debated whether biocrusts of different composition constitute different successional stages or different communities associated with microclimates. We think that, at least in our area of study, both models are true and compatible. There is a clear spatial pattern of distribution of biocrust communities according to their composition (Bevan, 2009). If we order the habitats in a microclimatic gradient and divide it into three zones, we would find the Cyanobacteria community in the most insolated places, because bacteria produce more photo-protective pigments than lichens (Miralles et al., 2017) and their metabolic response is faster when they get wet (Lange, 2001), which allows them to take advantage of smaller water inputs that take less time to evaporate. In the broad center of the gradient, we would find the most widespread lichen biocrusts, dominated by Squamarina and/or Diploschistes communities, and these lichens have been observed (Lázaro et al., 2008) to replace or cover a pre-existing microbiotic crust. At the shadiest extreme of the gradient, we would find the most stenotic and demanding species, with slower development: the Lepraria community, a habitat suitable for all the organisms studied, which allows it to host the complete succession eventually.

We do not mean that the concrete communities studied here follow or replace each other exactly. They represent each of stage of the succession process. Abstractions are recognizable but variable in time and space, and they are compatible with the fact that succession does not follow the same path at different points due to the high number of local factors influencing it. Despite all this, as Lázaro et al. (2023) say, succession is the best conceptual model to understand aspects of biocrust dynamics. Thus, a certain biocrust can be considered a community associated with a microhabitat and a successional stage simultaneously.

Another result to be highlighted is that the recovery of the biocrusts does not adequately adjust to a linear function with time. This is relevant, considering that most of the recovery articles, in addition to covering only a short time period, estimate the biocrust recovery time from a linear extrapolation of their data. In 2001, Belnap and Eldrige highlighted this problem and observed that linear extrapolation of the data overestimated the biocrust recovery time. Based on our results (Table 1), we think that the recovery of the biocrust is more adequately described mainly by the sigmoidal function. This is clear from the growth functions fits of the microbial crust, especially in the Cyanobacteria community, fully recovered, and is aligned with other authors’ results (Kumpula et al., 2000; Xiao et al., 2019). Unlike the linear function, the sigmoidal function assumes a relatively slow initial phase and then an almost linear growth that slows down as it approaches a maximum at which it remains constant due to limited space or available resources. In arid and semiarid zones, the most frequent limiting factor is low water availability (Maestre et al., 2016). Although the results do not show it, we think that the lichen crust recovery probably fits a sigmoidal function as well. The frequent lack of water limits the speed of lichen growth and, despite being able to compete for space with vascular vegetation in areas with less available water, growth is also limited due to the competition between lichen species. The lack of adjustment to the sigmoidal function and to any of those analyzed (R^2^ was lower than for microbial crust) seems to be an indicator of the relatively early stage of development of these recovering lichen communities. This can be seen in Figure 2B, in which the lichen coverage of the disturbed areas is far from the cover of the control areas of these communities (about 45–50% with respect to the controls). The frequently unsuccessful colonization attempts, together with their growth rate being slower than that of the microbial crust, mean that the lichen crust recovers more slowly and, at present, is in the approximately lineal part of the sigmoidal function, due to which it does not adjust to that curve simultaneously with the microbial crust. In addition, from Figure 2, it can be observed that the development of biocrust (when adding the coverings of microbial crust and lichen crust) does not increase steadily over time until reaching a maximum. This could have multiple causes, such as oscillations in microclimate and availability of resources, species replacement, or trampling by animals throughout the years. Although the long field experience and even the Figure 1 suggest that almost all the lichen cover should be a replacement cover, we cannot rule out that some lichens developed directly on bare soil. To determine replacement rates of microbial crust by lichen crust requires further research at cell-scale.

Predicting the final recovery time of the biocrust from the sigmoidal function equations requires the assumption of a certain cover value as the final cover, as well as a constant increase in biocrust cover during recovery. The cover of undisturbed (control) areas decreased over time (Table 2) due to internal and external factors, and the current cover value provides the reference to decide when recovery has finished. However, the fluctuations in microbial and lichen crust cover during the recovery (Figure 2), the lack of full recovery of the lichen crust, and the low R^2^ to sigmoid function prevent reliable predictions, except for microbial biocrusts. These facts possibly also explain the differences in the estimates of the recovery times from previous works (Eldridge and Ferris, 1999; Belnap and Eldridge, 2001; Belnap and Warren, 2002; Lalley and Viles, 2008), in addition to the type and severity of disturbance, size of the disturbed surface, and the climatic and edaphic conditions of the disturbed areas (Belnap and Eldridge, 2001; Weber et al., 2016). Although we cannot establish reliable recovery rates, our results show that the microbial crust has already recovered in all the communities; it has grown by around 5 or 6% per year (annual average of increases and decreases in cover). The lichen crust, still to be recovered, shows and average net growth of around 0.7% per year in Lepraria and 1.7% per year in Squamarina and Diploschistes. Differences between types of crust agree with what was obtained in other works (Belnap and Warren, 2002; Dojani et al., 2011; Gypser et al., 2015; Williams et al., 2018, Lorite et al., 2020, among others). The recovery of the microbial crust ensures a certain stabilization of the soil, which favors the establishment of lichens (Belnap and Büdel, 2016; Deng et al., 2020), reduces erosion (Chamizo et al., 2012a) and reduces the risk of degradation of these areas (Belnap, 1995; Cantón et al., 2021).

Differences in the colonizing capacity of microbial and lichen crusts are also observed from the ring analysis (Figure 3). The results reveal higher recovery rates of lichen crust on the outer perimeters of the plots in lichen-dominated communities, except in the Diploschistes community, where it recovered first in the central ring. This is consistent with observations by Williams et al. (2018). They analyzed the recovery of previously scalped biocrust along a latitudinal gradient and observed that in Austrian and Spanish plots, biocrust colonization was higher at the edges than in the center of the plot. The greater stability of the undisturbed areas, mainly covered with lichens, seemed to favor the successful colonization of nearby areas—in our case, the outer ring—as well as the growth of lichens in these undisturbed areas. Only some pioneer species, such as *Fulgensia fulgida*, *Fulgensia desertorum*, or *Endocarpon pusillum*, usually small, can successfully colonize the central areas of the plots, as observed in the Cyanobacteria community (Figure 3). On the contrary, based on the results (Figure 3), the microbial community did not seem to be influenced by the undisturbed perimeter and had no difficulties colonizing any part of the plot rapidly.

Regarding the effect of microclimate oscillations on recovery, according the PCAs, the positions of the microbial and lichen crust covers in the microclimatic space diverge progressively, as the involved lichen species are expected to be more stenoic, or demanding of resources (Figure 6). The lichen crust position in the PCA biplot of Lepraria (Figure 6D) strongly suggests that in this community new lichen species are replacing or dominating to the previous ones. On the other hand, the microclimatic effect on recovery seems to be different for each community. In Cyanobacteria, both microbial and lichen crust cover are associated with potential dew (Figure 6A); however, there is less dew but more water vapor adsorption according to Uclés et al. (2015) and Lopez-Canfin et al. (2022), who show that the absorbed vapor is condensed within the soil, benefiting the microbial crust. In the late-successional Lepraria community, lichen crust cover is clearly associated with the variables that indicate greater water availability, except for potential dew, which is associated with lichen growth in the other communities. This lack of association does not mean there are fewer potential dew hours (in fact, we recorded in Lepraria greater number of hours). According to some authors dew is higher in communities dominated by lichen crust than in those dominated by microbial crusts (Cyanobacteria) (Uclés et al., 2015). But it seems that recovery in Lepraria is more associated with longer periods of humidity. In the early and mid-successional communities (except in Squamarina, because the most widespread lichen species does not seem to be related to any variable) lichen growth is much more associated with potential dew and PAR, and not with parameters related to large water inputs. However, the regressions between microclimatic variables and covers were not significant, probably because, while microclimatic oscillations (responsible for processes such as net photosynthesis) occur at much finer time scales, the species cover data were only annual. The cover after one year depends little on, for example, the total precipitation at the end of the year, and more on when and how that precipitation occurred. Other reasons these regressions might not be significant, even though the influence of the microclimate on the recovery of the biocrust was real [as suggested by Lázaro et al. (2008)], are that (i) the microclimate could particularly affect the initial phases of recovery, as proposed by Williams et al. (2018); (ii) the effects could change depending on the evolution of the specific composition; such a change is to be expected, considering that different communities are adapted to somewhat different microclimates, which could blur the effects when considering a long enough time frame to capture changes in species composition; and (iii) the climatic differences between years might not be large enough to significantly influence the recovery of the biocrust, which changes slowly. On the other hand, Harper and Marble (1988) stated that dry-season disturbances are more destructive than wet-season disturbances. The effects of timing of disturbance (in the context of the annual climate cycle) are also important in the early years of recovery, as observed by Dojani et al. (2011) and Hawkes and Flechtner (2002), who removed an area of shoreline just before the wet season. Considering this, the frequency and intensity of the rainiest years in a series could also modify the recovery results. Moreover, although developed biocrust resists rain splash (Lázaro et al., 2023), the rain intensity (not considered here) is very important because it could remove the incipient lichen thalli, explaining the failed colonization attempts observed during the inventories. Thus, for the same total annual rainfall, the timing of the rain events is important as well. After this research, we realized that the study of the relationships between microclimate oscillations and the dynamics of biocrusts is just starting, as these relationships are complex and the cover data refer necessarily to a much rougher time scale than the actual biocrust’s photosynthetic and respiratory activities, showing the effect of microenvironments in carbon assimilation. In addition, the cover at any time is the result of a sequence of increasing and decreasing phases, which makes it difficult to determine the real effect of the microclimate on the development of biocrusts if the cover is not recorded continuously or, at least, seasonally.

## Conclusions

Our results provide quite a bit of new information about microbial and lichen colonization and biocrust recovery in semiarid regions after a mechanical disturbance by removal, because this research is based on a longer series than usual. We also propose a new point of view of the recovery of biocrusts, analyzing how the recovery process works throughout time and what factors influence it. Biocrust recovery is well described by a sigmoidal function in the microbial crust; we argue that the lichen crust development will have the same shape, but to verify this would requires a longer data series. The total time needed for recovery of the biocrust can be long, but it depends on the specific composition of the biocrust, as well as on the proximity and composition of the neighboring communities and the microclimatic characteristics of the area. We cannot reliably estimate recovery time with the current data, except for the Cyanobacteria community, which was fully recovered after 13 years.

Biocrusts’ composition stabilizes quite quickly when the disturbed area is small. The frequency of failed colonization attempts seems to decrease over the years, as the microbial crust spreads and lichens colonize on it and not directly on the bare soil, which has much less cohesion, so the small thallus detaches easily. Lichen colonization represents feedback in biocrust stabilization and biodiversity even before it fully recovers, decreasing erosion.

Although the influence of water availability on biocrust recovery is not apparent on large time scales, except for Lepraria community, a great deal of evidence points to water availability being important for the development of lichens and mosses at monthly to hourly time scales. Dew seems to be quite important for more exposed communities with less (intermediate) water availability and our own unpublished studies confirm that prolonged lack of water can hinder the growth of lichens and mosses. The challenge now is to find methods linking the growth of various biocrusts with microclimatic variables, as the microclimatic oscillations affecting metabolic activity are much faster than the visible cover changes they produce.

Our results support the hypothesis of a succession characterized by three phases that can be represented by the studied communities: a first colonization of the microbial crust, dominated by Cyanobacteria, followed by an extensive development of lichens, and later development of more shady lichens and mosses, which requires that other organisms change the preconditions of the microhabitat to develop. The succession may not be obvious, because it runs at different speeds in different places and almost never occurs in the most stressing habitats; thus, the communities that characterize the driest and the medium habitats can appear to be permanent. In addition, succession is influenced by climatic, geomorphological, and resource constraints.

## Data availability statement

The raw data supporting the conclusions of this article will be made available by the authors, without undue reservation.

## Author contributions

RL conceived the idea, designed the experiment, and start the inventories. CR contributed to the inventories for the last three years and processed and analyzed the data, and wrote the first version of the text. RL and CR wrote the final version. Both authors contributed to the article and approved the submitted version.
